# Physiological and Ecological Responses of Arctic and Alpine Plants to Climate Change: Integrating Mechanisms, Adaptations and Ecosystem Consequences

**DOI:** 10.3390/plants15142105

**Published:** 2026-07-08

**Authors:** Guoyan Wang, Peili Shi

**Affiliations:** 1 State Key Laboratory of Geohazard Prevention and Geoenvironment Protection, Chengdu University of Technology, Chengdu 610059, China; 2 Institute of Geographic Sciences and Natural Resources Research, Chinese Academy of Sciences, Beijing 100101, China

## 1. Introduction

Alpine and arctic ecosystems are undergoing some of the most rapid environmental changes on the Earth. Rising temperatures, altered precipitation regimes, changing snow cover, and increasing nitrogen deposition, combined with the growing frequency of extreme weather events, are reshaping plant physiological processes, species interactions, phenological dynamics, community assembly, and ecosystem functioning. As primary producers in alpine and arctic ecosystems, plants regulate biogeochemical cycles and energy flow; therefore, understanding their adaptive responses is critical for predicting ecosystem resilience [1,2,3].

Despite decades of research documenting large-scale vegetation shifts, understanding the underlying mechanisms, particularly the roles of physiological plasticity and stoichiometric regulation in mediating these responses remain a critical frontier [1,2,3,4,5]. Addressing these knowledge gaps requires a multi-faceted approach exploring how plants adjust their functional traits, resources strategies, and how altered phenology affects population persistence and the cascading impacts on biodiversity and ecosystem dynamics. This Special Issue, “Physiological and ecological responses of arctic and alpine plants to climate change,” assembles thirteen contributions (twelve research articles and one communication) that address these critical questions. The collected studies span scales from individual physiological responses to community and ecosystem-level processes, providing a comprehensive framework to understand the mechanisms of adaptative strategies and ecosystem consequences of climate induced stress.

## 2. Research Advances in Alpine and Arctic Plant Responses to Climate Change

### 2.1. Physiological and Functional Responses to Environmental Stress

Adaptive strategies in cold regions are dictated by physiological plasticity and resource allocation trade-offs. Liu et al. [1] demonstrate that interspecific variations in osmotic regulation, antioxidant activity, and hormonal signaling provide adaptive mechanism for maintaining dominant species performance, along elevational gradients. Zhao et al. [2] illustrate that functional trait divergence underpins the trade-off between water- and nitrogen-use efficiencies, linking species-level traits to ecosystem-level resource strategies. Zhao and Chen [6] highlight that desert shrubs in the Qaidam Basin coordinate water and nitrogen utilization via leaf trait adjustments, further emphasizing adaptive convergence under arid stress. Critically, Ma et al. [3] provide evidence that stoichiometric homeostasis and functional group divergence jointly enhance adaptation across environmental gradients. Together, these studies illustrate that physiological plasticity, trait-based resource allocation, and stoichiometric regulation interact to sustain plant performance under challenging environmental conditions.

### 2.2. Resource Acquisition and Plant–Soil Interactions

Plant–soil interactions emerge as critical mediators of adaptation. Yuan et al. [7] demonstrate that rhizosheath–mycorrhizal associations in *Kengyilia hirsuta* enhance phosphorus uptake, illustrating the importance of belowground symbioses in nutrient-limited habitats. Xing et al. [8] show that the cushion plant *Androsace tapete* modulates nitrogen acquisition of neighboring species, highlighting facilitation within alpine communities. At the microbial level, Chen et al. [9] report that nitrogen enrichment reshapes microbial networks differently in alpine meadows and steppes, reflecting ecosystem-specific responses. Huang et al. [10] find that plant functional group removal alters soil nematode communities and reduces soil particulate organic carbon, underscoring strong aboveground–belowground coupling. Collectively, these studies highlight that resource acquisition strategies are embedded within complex plant–soil–microbe networks, mediating ecosystem resilience.

### 2.3. Regeneration and Phenological Responses to Climatic Signals

Successful regeneration and phenological synchronization are vital for alpine plant persistence. Li et al. [11] reveal that seed wings in Smith fir (*Abies georgei* var. *smithii*) modulate germination timing in response to temperature and light, ensuring recruitment under optimal conditions. Zhang et al. [4] establish that snow dynamics significantly influence vegetation green-up across the Mongolian Plateau, highlighting the role of environmental cues in phenology. Together, these studies indicate that regeneration and phenology are tightly coupled to climatic signals, and shifts in these processes can have cascading effects on community and ecosystem dynamics.

### 2.4. From Community Assembly to Ecosystem Responses

Adaptive responses scale up from individual traits to dictate community composition and evolutionary trajectories. Li and Gheyret [12] analyze the patterns of ecological uniqueness across the Qinghai–Tibetan Plateau, identifying environmental and community-level drivers of biodiversity. Wang et al. [5] document climate-driven vegetation shifts and wetland expansion, illustrating landscape-scale impacts. He et al. [13] investigate diversification in *Meconopsis*, illustrating how life-history strategies and topographic heterogeneity drive evolutionary adaptation. These studies collectively demonstrate that adaptive strategies shape not only species persistence but also community composition and ecosystem processes.

### 2.5. Emerging Trends and Future Perspectives

Several priorities for future research emerge from this collection. Integrating across physiological, demographic, community, and ecosystem scales is essential for predictive ecology. Aboveground–belowground interactions, multifactorial environmental drivers, and trait-based, multi-omics approaches provide powerful tools to understand and forecast ecosystem dynamics. Long-term monitoring and mechanistic modeling will be critical to anticipate ecosystem trajectories [1,2,3,4,5,6,7,8,9,10,11,12,13]. As the guest editors, we warmly welcome contributions from polar regions to the forthcoming second edition of this Special Issue (https://www.mdpi.com/journal/plants/special_issues/2FW9V7NTRX (accessed on 8 June 2026)). Integrating evidence from both alpine and arctic systems will not only broaden the geographical scope of this research field but will also help identify shared adaptive strategies and ecological principles governing plant responses and ecosystem resilience across cold environments.

## 3. Concluding Remarks

This Special Issue provides a mechanistic understanding of how alpine and arctic plants respond to climate change. Physiological plasticity, resource-use strategies, plant–soil interactions, regeneration dynamics, biodiversity patterns, and ecosystem processes emerge as interconnected dimensions of ecosystem resilience. We thank all authors, reviewers, and the editorial team of *Plants* for their contributions. This collection aims to stimulate interdisciplinary research and inform the conservation and management of cold-region ecosystems in a changing world.
